# Urinary Biomarkers of Inflammation and Oxidative Stress Are Elevated in Obese Children and Correlate with a Marker of Endothelial Dysfunction

**DOI:** 10.1155/2019/9604740

**Published:** 2019-10-16

**Authors:** Vaithinathan Selvaraju, Priscilla Ayine, Moni Fadamiro, Jeganathan Ramesh Babu, Michael Brown, Thangiah Geetha

**Affiliations:** ^1^Department of Nutrition, Dietetics & Hospitality Management, Auburn University, AL, USA; ^2^Boshell Metabolic Diseases and Diabetes Program, Auburn University, Auburn, AL, USA; ^3^School of Kinesiology, Auburn University, AL, USA

## Abstract

Obesity is a state of chronic low-level inflammation closely associated with oxidative stress. Childhood obesity is associated with endothelial dysfunction, inflammation, and oxidative stress markers individually. This study was aimed at determining the association between the biomarkers of inflammation, oxidative stress, and endothelial dysfunction in urine samples of healthy, overweight, and obese children. Eighty-eight elementary school children aged between 6 and 10 years participated in this study. Anthropometric measurements were measured using WHO recommendations. The biomarkers of low-grade inflammation such as C-reactive protein (CRP), interleukin-6 (IL-6), and *α*-1-acid glycoprotein (AGP); oxidative stress markers such as 8-isoprostane and 8-hydroxy-2′-deoxyguanosine (8-OHdG); and endothelin-1 (ET-1) were analyzed in urine samples. The area under the curve (AUC) by the receiver operating characteristics (ROC) was analyzed to identify the best urinary biomarker in childhood obesity. Linear regression and Pearson correlation were analyzed to determine the association between the parameters. The obese participants have significantly increased levels of CRP, AGP, IL-6, and 8-isoprostane compared to normal-weight participants. The overweight participants had significantly increased levels of ET-1 and 8-OHdG but not the obese group compared to the NW group. The AUC for urinary CRP (AUC: 0.847, 95% CI: 0.765-0.930; *p* < 0.0001) and 8-isoprostane (AUC: 0.857, 95% CI: 0.783-0.932; *p* < 0.0001) showed a greater area under ROC curves compared to other inflammatory and oxidative markers. The urinary CRP and 8-isoprostane significantly correlated with the obesity measures (body mass index, waist circumference, and waist-to- height ratio) and ET-1, inflammatory, and oxidative markers. The increased urinary inflammatory markers and 8-isoprostane can serve as a noninvasive benchmark for early detection of the risk of developing cardiovascular disease.

## 1. Introduction

Obesity is a major health problem with increasing rates in adults and children worldwide [1]. In the United States, around 13.7 million children and adolescents are obese [2]. In 2015, 35% of children in Alabama are overweight and obese, ranked 6th highest with obesity in the United States [3]. Obesity increases the risk of developing cardiovascular diseases, diabetes, musculoskeletal disorders, asthma, sleep disorders, and some cancers. Obesity results when there is a higher accumulation of body fat that could affect normal health [4]. Childhood obesity has linked with a higher chance of obesity in adulthood, reduced lifespan, premature death, and increased metabolic complications, including cardiovascular disease [5–7].

Inflammation and oxidative stress are important factors in the development of cardiovascular disease [8–11]. The metabolic risk factors develop at an early age; endothelial dysfunction is induced before any symptoms appear for atherosclerosis [12–14]. The enlargement of adipocytes by fat deposition in obese individuals caused adipose tissue hypoxia and increased secretion of inflammatory cytokines. High levels of serum inflammatory cytokines and markers such as tumor necrosis factor-*α* (TNF-*α*), interleukin-6 (IL-6), C-reactive protein (CRP), and alpha-1-acid glycoprotein (AGP) have been correlated with the endothelial dysfunction [15]. Obesity is a state of chronic low-grade inflammation and observed with endothelial dysfunction that could lead to atherosclerosis [16–19].

Oxidative stress also plays a significant role in inducing endothelial dysfunction [20], which could lead to atherogenesis [21]. The production of reactive oxygen species (ROS) is increased in obesity due to increased micronutrient intake, metabolic production, mitochondrial dysfunction, and endoplasmic reticulum stress [22]. An enhanced metabolic rate is linked with obesity [23]. The increased metabolic rate is correlated with higher production of ROS, thereby leading to increased lipid peroxidation and oxidative injury [24]. Increased body mass index (BMI) during obesity is also found to be an independent risk factor for high levels of oxidative stress [25]. Increased oxidative stress is more likely to induce vascular damage in obese children [26].

Childhood obesity has been associated with endothelial dysfunction [27], inflammation [28], and oxidative stress [22, 29] markers independently; however, the interrelation between these factors is unknown. The objective of this study is to determine the interrelationship of inflammation, oxidative stress, and endothelial dysfunction in urinary samples to enhance the understanding of the childhood obesity risks in the pathogenesis of cardiovascular disease. The levels of urinary endothelin-1; inflammatory biomarkers such as IL-6, CRP, and AGP; and oxidative stress markers, namely, 8-isoprostane and 8-OHdG, were measured in normal, overweight, and obese children. The receiver operating characteristic (ROC) analysis was used to determine the area under the curve (AUC) to identify the best urinary biomarker in children. The linear regression and Pearson correlation analyses were used to determine the association between the biomarkers. This study will provide a basis for noninvasive screening and monitoring in children.

## 2. Materials and Methods

### 2.1. Study Population

This study was approved by the Auburn University Institute Review Board (IRB), and written parental/participant consent form was collected from all participants before obtaining the samples. Based on an initial phone survey with the parents, children with a history of diabetes or cardiovascular disease were excluded. Eighty-eight children were recruited from Lee County and Macon County, Alabama, with ages ranging from 6 to 10 years.

### 2.2. Anthropometric Measurements

Participant anthropometric measurements were collected as per the World Health Organization (WHO) recommendations. Body weight was recorded without shoes, only with light clothing (nearest 0.1 kg) using a Tanita digital scale (WB-800H plus) [30], and height was measured to the nearest 0.1 cm using a stadiometer attached to the scale. Based on the height and weight, BMI was calculated according to the Centers for Diseases Control and Prevention (CDC) growth chart. The measured BMI was used to classify the participants as underweight (<5^th^ percentile), normal weight (≥5^th^ to ≤85^th^ percentile), overweight (>85^th^ to ≤95^th^ percentile), and obese (>95^th^ percentile) [31]. Since the growth will occur until the age of twenty, BMI *z*-scores are calculated using SPSS macro based on WHO growth reference 2007 data, which were adjusted for age and sex [32]. Fat distribution was evaluated by measuring the waist circumference (WC). The *z*-score for WC and the ratio of waist to height (WHtR) was calculated using the R macro package developed by Sharma et al., based on LMS tables from NHANES III [33].

### 2.3. Measurement of Urinary Biomarkers of Low-Grade Inflammation, Endothelin-1, and Oxidative Stress

The urine sample was collected from the participants during screening in the daytime in a sterile urine sample collection container. The sample was aliquoted in 15 ml falcon tubes and centrifuged at a speed of 2000 rpm for 10 min at 4°C to precipitate the particulates. The centrifuged urine samples were stored at -80°C as aliquots to avoid the freeze/thaw cycle until further usage. The level of urinary inflammatory biomarkers such as IL-6, CRP, and AGP was determined by ELISA (R&D Systems, MN, USA) in duplicate according to the manufacturer's instructions. Endothelin-1 (R&D Systems, MN, USA) and the oxidative stress markers, namely, 8-isoprostane (Abcam, MA, USA) and 8-OHdG (R&D Systems, MN, USA), were also analyzed by ELISA. The concentration of all the biomarkers was normalized with the corresponding urinary creatinine levels (R&D Systems, MN, USA). Detailed methodology of the ELISA assay has been provided in supplementary materials ([Supplementary-material supplementary-material-1]).

### 2.4. Statistical Analysis

The sample size was calculated using the free power and sample size calculation (PS) software version 3.1.6 with greater than 80% power with a type 1 error probability of 0.05 to identify a logical difference in the biomarkers and association analysis between the groups. The calculated sample size was verified in the sample size requirement table [34]. All the calculation, along with normalization of creatinine, was performed in an Excel sheet and maintained. Statistical analysis was performed by GraphPad Prism (5.0, GraphPad Software, CA, USA) for the one-way ANOVA test for three-group comparison. Tukey's test was used for post hoc comparison of means between each pair of groups. The results are expressed as mean ± standard error. The concentration of the biomarkers was plotted with 5-95% box whisker plots. Variables (CRP, IL-6, AGP, ET-1, 8-isoprostane, and 8-OHdG) with skewed distributions were natural logarithm transformed by SPSS (version 24, IBM, Armonk, NY, USA), and the following analyses were performed. The receiver operating characteristic (ROC) curve analysis was performed to determine the diagnostic accuracy of the different biomarkers of obesity. The area under the curve (AUC) was calculated, and it distinguishes the diagnostic value of the biomarkers. The value of the area under the curve (AUC) ranges between 1 (perfect test) and 0 (worthless test). Pearson correlation and linear regression analyses were done to determine the association between various parameters. *p* < 0.05 was considered statistically significant.

## 3. Results

The general characteristics of the study participants are shown in Table 1. Eighty-eight participants (41—normal-weight (NW), 24—overweight (OW), and 23—obese (OB) children) aged between 6 and 10 years were included in this study. The mean age and height of the participants were not statistically different between the groups (Table 1). However, the anthropometric measurements such as body weight (34.42 ± 1.31 kg and 43.77 ± 2.06 kg), BMI (19.25 ± 0.22 kg/m^2^ and 23.31 ± 0.58 kg/m^2^), and BMI *z*-score (1.11 ± 0.10 and 2.21 ± 0.14) of OW and OB were significantly (*p* < 0.0001) increased compared to those of NW participants (weight—29.90 ± 1.07 kg, BMI—16.38 ± 0.20 kg/m^2^, and BMI *z*-score—0.06 ± 0.08). Body fat distribution or accumulation was evaluated by waist circumference in OW and OB subjects; the WC (67.46 ± 1.27 cm and 76.02 ± 1.66), WC *z*-score (1.00 ± 0.08 and 1.60 ± 0.07), and waist-to-height ratio (WHtR) *z*-score (1.24 ± 0.09 and 1.72 ± 0.16) were significantly greater (*p* < 0.0001) compared to those of NW subjects (WC—61.28 ± 0.70 cm, WC *z*-score—0.17 ± 0.07, and WHtR *z*-score—−0.03 ± 0.09).

**Figure 1:**
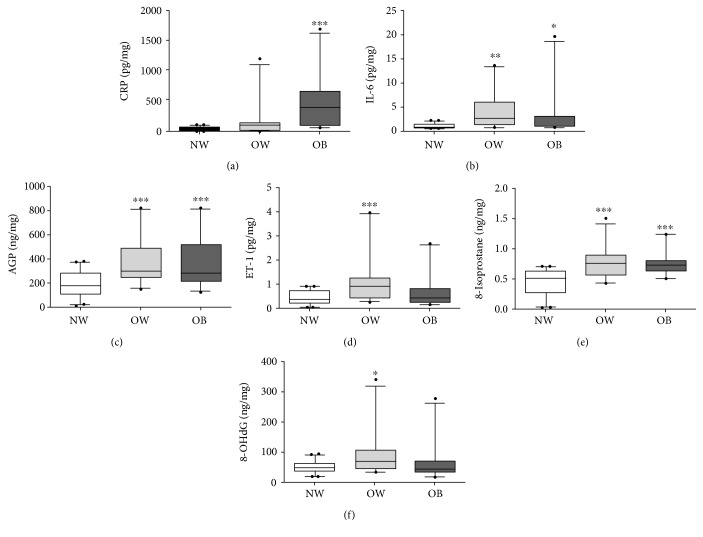
Comparison of urinary biomarkers in normal-weight, overweight, and obese participants. Urinary expression of median (min–max) and lower and upper quartile is represented as box and whisker plots: (a) CRP, (b) IL-6, (c) AGP, (d) ET-1, (e) 8-isoprostane, and (f) 8-OHdG. ^∗^*p* < 0.05, ^∗∗^*p* < 0.01, and ^∗∗∗^*p* < 0.0001 compared to normal weight (*n* = 41, NW; *n* = 24, OW; and *n* = 23, OB).

To investigate the differences in the inflammatory biomarkers in healthy, overweight, and obese children, we measured CRP, IL-6, and CRP in urine samples. The sensitive systemic inflammatory marker CRP increased in OW (157.7 ± 55.47 pg/mg) and significantly increased in obese (476.4 ± 99.06 pg/mg; *p* < 0.0001), in comparison to NW (32.4 ± 4.18 pg/mg) as shown in Figure 1(a). We observed a significant increase in IL-6 concentration in OW (3.78 ± 0.74 pg/mg; *p* < 0.002) and obese (2.94 ± 0.96 pg/mg; *p* < 0.043), compared to NW participants (1.01 ± 0.10 pg/mg) (Figure 1(b)). The AGP is one of the acute-phase proteins that showed a significant increase in the OW (363.5 ± 37.64 ng/mg; *p* < 0.0001) and obese (358.3 ± 41.55 ng/mg; *p* < 0.0001) group in comparison with NW (191.1 ± 19.89 ng/mg) (Figure 1(c)). The endothelin family protein and inflammatory mediator ET-1 was significantly increased in OW (1.10 ± 0.20 pg/mg; *p* < 0.0001) and was increased in obese participants (0.63 ± 0.14 pg/mg) but not significantly compared to NW (0.43 ± 0.05 pg/mg) (Figure 1(d)).

We also found the nonenzymatic oxidative stress marker 8-isoprostane levels were significantly higher in the OW (0.78 ± 0.05 ng/mg; *p* < 0.0001) and obese (0.77 ± 0.04 ng/mg; *p* < 0.0001) group in comparison to NW (0.45 ± 0.04 ng/mg) urine samples (Figure 1(e)). However, there was statistically significant difference in the nonenzymatic stable end-product of DNA oxidation, 8-hydroxy-2′-deoxyguanosine (8-OHdG), among the OW group (86.19 ± 14.69 ng/mg; *p* < 0.01), the obese group (64.24 ± 13.1 ng/mg), and the NW group (47.39 ± 2.89 ng/mg) as shown in Figure 1(f).

**Figure 2:**
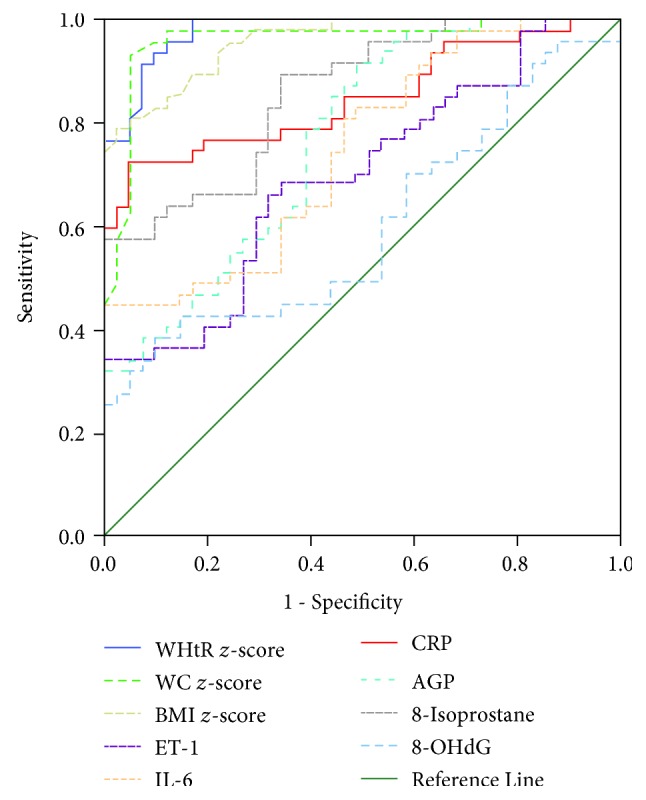
Receiver operating characteristic (ROC) curve showing the performance of anthropometric measures and urinary IL-6, CRP, AGP, ET-1, 8-isoprostane, and 8-OHdG.

ROC with the area under the curve (AUC), cut-off, sensitivity, and specificity were determined to analyze the predictive value of the obesity measures and urinary markers. The AUC values (with 95% confidence interval (CI)) of the anthropometric measurements are shown as follows: BMI *z*-score (AUC: 0.957, 95% CI: 0.922-0.992; *p* < 0.0001), WC *z*-score (AUC: 0.960, 95% CI: 0.917-1.000; *p* < 0.0001), and WHtR *z*-score (AUC: 0.978, 95% CI: 0.956-1.000; *p* < 0.0001). Among the inflammatory and oxidative stress markers, the AUC for CRP (AUC: 0.847, 95% CI: 0.765-0.930; *p* < 0.0001) and 8-isoprostane (AUC: 0.857, 95% CI: 0.783-0.932; *p* < 0.0001) were the highest compared to those for IL-6, AGP, ET-1, and 8-OHdG as shown in Figure 2 and Table 2. ROC results show the best cut-off value for CRP was 72.65 pg/mg for the diagnosis of urinary markers and the oxidative stress marker 8-isoprostane cut-off value was found to be 0.58 ng/mg (*p* < 0.0001) with sensitivity and specificity.

**Figure 3:**
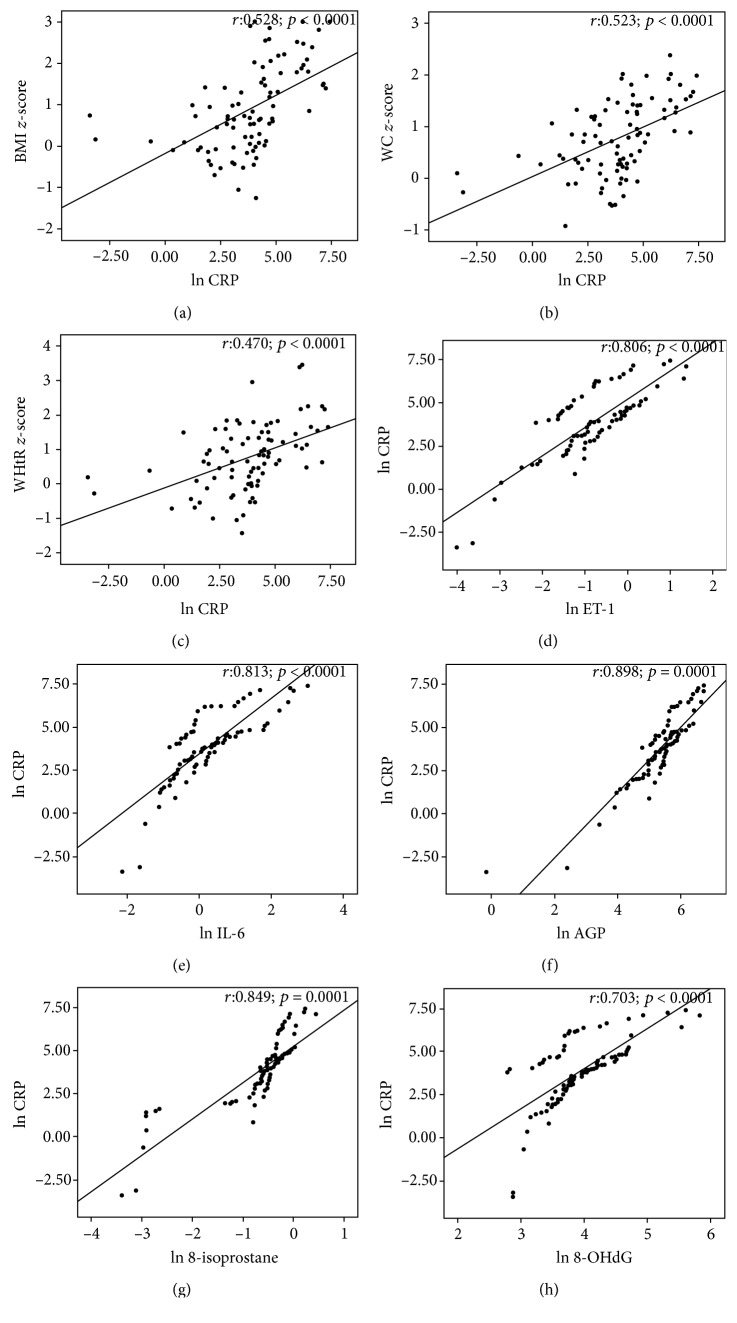
Correlation of the urinary inflammatory marker CRP with anthropometric measurements (a) BMI *z*-score, (b) WC *z*-score, and (c) WHtR *z*-score and ET-1, IL-6, and AGP (d–f) with oxidative stress markers 8-isoprostane and 8-OHdG (g–h) (*n* = 41, NW; *n* = 24, OW; and *n* = 23, OB).

The inflammatory marker CRP is positively (*p* < 0.0001) associated with BMI *z*-score (*r* = 0.528), WC *z*-score (*r* = 0.523), and WHtR *z*-score (*r* = 0.470) as shown in Figures 3(a)–3(c). Assessment of CRP correlation with other inflammatory markers such as ET-1 (*r* = 0.806; *p* < 0.0001), IL-6 (*r* = 0.813; *p* < 0.0001), and AGP (*r* = 0.898; *p* < 0.0001) and oxidative stress markers 8-isoprostane (*r* = 0.849; *p* < 0.0001) and 8-OHdG (*r* = 0.703; *p* < 0.0001) showed a significant positive association (Figures 3(d)–3(h)).

**Figure 4:**
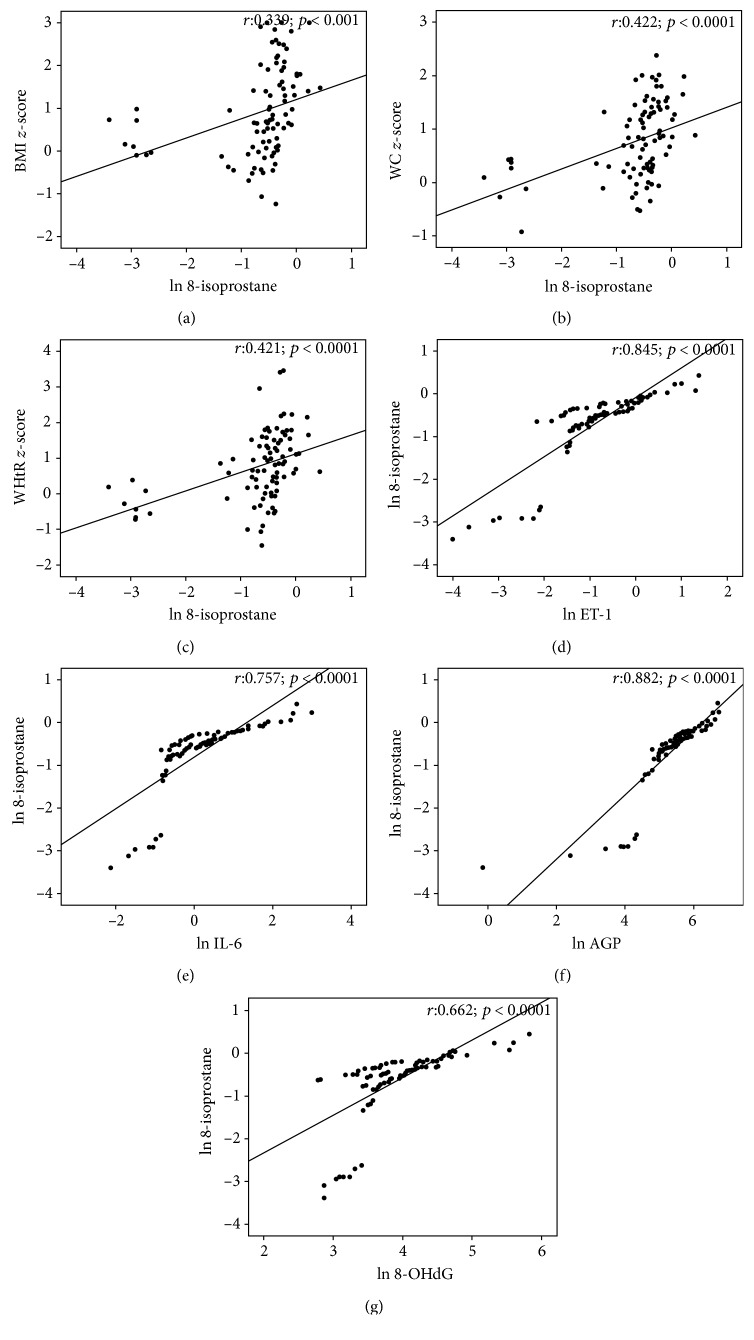
Scatter plot for correlation analyses of the oxidative stress marker 8-isoprostane with (a) BMI *z*-score, (b) WC *z*-score, and (c) WHtR *z*-score. (d–e) The significant association of 8-isoprostane with inflammatory markers and ET-1 (*n* = 41, NW; *n* = 24, OW; and *n* = 23, OB).

We also observed the significant positive association of the oxidative stress marker 8-isoprostane with the obesity measurements, BMI *z*-score (*r* = 0.339; *p* < 0.001), WC *z*-score (*r* = 0.422; *p* < 0.0001), and WHtR *z*-score (*r* = 0.421; *p* < 0.0001) (Figures 4(a)–4(c)). Analysis was done to see whether there is an association between 8-isoprostane and inflammatory markers. As shown in Figures 4(d)–4(g), 8-isoprostane showed a significant positive correlation with ET-1 (*r* = 0.845; *p* < 0.0001), IL-6 (*r* = 0.757; *p* < 0.0001), and AGP (*r* = 0.882; *p* < 0.0001) and also with 8-OHdG (*r* = 0.662; *p* < 0.0001). Pearson correlation analysis data for anthropometric measurements and urinary markers are provided in Table 3, and all the data obtained are positively correlated.

## 4. Discussion

This study suggests that obese children have significantly increased levels of urinary CRP, IL-6, AGP, ET1, and 8-isoprostane compared to normal-weight children. The ET-1 and 8-OHdG significantly increased in the overweight group and markedly increased in the obese group. These findings are reliable with other previous studies demonstrating obesity associated with increased inflammation and oxidative stress [15, 25, 35]. The levels of some of these urinary markers are significantly high in the overweight group and not in the obese group compared to the normal-weight group. The reason for the difference in the expression of these markers needs to be further studied. The ROC curve analysis of all the obesity measures such as BMI, waist circumference, and waist-to-height ratio *z*-score has AUC greater than 0.90 and is considered an excellent diagnostic measurement to determine obesity. The AUC for CRP (AUC: 0.847, 95% CI: 0.765-0.930; *p* < 0.0001) was greater than those for other urinary inflammatory biomarkers. Similarly, urinary 8-isoprostane (AUC: 0.857, 95% CI: 0.783-0.932; *p* < 0.0001) had greater AUC compared to 8-OHdG. The important aspect of this study is to demonstrate that urinary CRP and 8-isoprostane showed a positive correlation with obesity measurements and inflammatory, oxidative stress, and endothelial dysfunction markers. We further analyzed the association of CRP and 8-isoprostane with the obesity measures and other inflammatory, oxidative stress, and endothelial dysfunction markers. CRP was significantly (*p* < 0.0001) associated with all the three obesity measures such as BMI *z*-score (*r* = 0.528), WC *z*-score (*r* = 0.523), and WHtR *z*-score (*r* = 0.470). Also, the urinary CRP significantly (*p* < 0.0001) positively correlated with the endothelial dysfunction marker ET-1, inflammatory markers IL-6 and AGP, and oxidative stress marker 8-OHdG. BMI *z*-score (*p* < 0.001), WC *z*-score (*p* < 0.0001), WHtR *z*-score (*p* < 0.0001), and other urinary (ET-1, IL-6, AGP, and 8-OHdG) markers (*p* < 0.0001) also significantly correlated with the oxidative stress marker 8-isoprostane.

Early detection of endothelial dysfunction will help to detect the risk of cardiovascular morbidity [12–14]. The endothelial function assessment is generally done by brachial artery flow-mediated dilation (FMD) [36]. The analysis of FMD needs an evaluation of endothelial-independent vasodilation of glyceryl nitrate. In children and adolescents, the study of nitrate-dependent vasodilation was excluded due to legal and ethical issues [37, 38]. FMD has shown to decrease in obese children with insulin resistance [37, 39], hypertension [40], and dyslipidemia [41]. FMD is lower in obese children [38, 42], and inflammation and oxidative stress factors may be involved in endothelial dysfunction.

In our study, 8-OHdG, the product of oxidative DNA damage, was not elevated in obese children but correlated with CRP and 8-isoprostane. The level of 8-OHdG in urine determines the DNA damage in the whole body [43]. It is a marker to evaluate cardiovascular disease (CVD) and inflammatory status in patients with hypertension [44, 45]. It also helps to identify the risks of cancer and early detection associated with lifestyle-related diseases [43, 46]. However, the urinary 8-isoprostane level significantly increased with obesity measures and correlated with inflammatory markers and ET-1. Isoprostanes are increased due to oxidative lipid damage and excreted in urine due to nonenzymatic peroxidation of arachidonic acid [47]. It is an indication of lipid peroxidation and the gold standard for oxidative stress and damage [48]. The results are based upon the small number of participants, and this needs to be confirmed with a larger cohort study. The major advantage of this study is the use of a noninvasive urine sample, specifically in children.

## 5. Conclusion

As obesity is a state of chronic low-level inflammation closely associated with oxidative stress. The association of biomarkers of inflammation, oxidative stress, and endothelial dysfunction in urine samples of healthy and obese children has been the focus of this study. The level of urinary CRP and 8-isoprostane significantly increased as the obesity measures increase and correlated with ET-1, inflammatory, and oxidative stress markers. The increased urinary inflammatory markers and 8-isoprostane can serve as a noninvasive benchmark for early detection of the risk of developing cardiovascular disease.

## Figures and Tables

**Table 1:** Anthropometric characterization of the study population.

Parameter	NW	OW	OB	*p* value
Sex (*N* = 88)	41	24	23	—
Male/female (46/42)	23/18	14/10	9/14	—
Age (years)	8.83 ± 0.21	8.44 ± 0.25	8.54 ± 0.26	NS
Height (cm)	134.18 ± 1.82	133.08 ± 1.91	136.50 ± 1.96	NS
Weight (kg)	29.90 ± 1.07	34.42 ± 1.31	43.77±2.06^∗∗∗^	*p* < 0.0001
BMI (kg/m^2^)	16.38 ± 0.20	19.25±0.22^∗∗∗^	23.31±0.58^∗∗∗^	*p* < 0.0001
BMI *z*-score	0.06 ± 0.08	1.11±0.10^∗∗∗^	2.21±0.14^∗∗∗^	*p* < 0.0001
Waist circumference (cm)	61.28 ± 0.70	67.46±1.27^∗∗∗^	76.02±1.66^∗∗∗^	*p* < 0.0001
WC *z*-score	0.17 ± 0.07	1.00±0.08^∗∗∗^	1.60±0.07^∗∗∗^	*p* < 0.0001
WHtR *z*-score	−0.03 ± 0.09	1.24±0.09^∗∗∗^	1.72±0.16^∗∗∗^	*p* < 0.0001

^∗∗∗^ *p* values were compared to the normal-weight (NW) group. NS: not significant.

**Table 2:** ROC curve analysis of anthropometric measures and urinary biomarkers shows the relationship between sensitivity and specificity in determining a specific marker.

Parameter	AUC	SE	Cut-off	Sensitivity	1 − specificity	Significance *p* value	95% CI	
							Lower	Upper
BMI *z*-score	0.957	0.018	0.695	0.851	0.122	<0.0001	0.922	0.992
WC *z*-score	0.960	0.022	0.705	0.936	0.049	<0.0001	0.917	1.000
WHtR *z*-score	0.978	0.011	0.670	0.915	0.073	<0.0001	0.956	1.000
ET-1	0.695	0.055	0.437	0.660	0.317	<0.002	0.587	0.804
IL-6	0.747	0.051	0.837	0.809	0.463	<0.0001	0.646	0.847
CRP	0.847	0.042	72.65	0.723	0.049	<0.0001	0.765	0.930
AGP	0.769	0.050	222.22	0.787	0.390	<0.0001	0.671	0.866
8-Isoprostane	0.857	0.038	0.575	0.894	0.341	<0.0001	0.783	0.932
8-OHdG	0.601	0.061	66.45	0.426	0.146	<0.104	0.482	0.720

**Table 3:** Pairwise Pearson correlation between salivary obesity biomarkers and anthropometric parameters.

Parameters	ET-1		IL-6		CRP		AGP		8-Isoprostane		8-OHdG	
	*r*	*p*	*r*	*p*	*r*	*p*	*r*	*p*	*r*	*p*	*r*	*p*
BMI *z*-score	0.166	<0.122	0.301	<0.004	0.528	<0.0001	0.294	<0.006	0.339	<0.001	0.111	<0.305
WC *z*-score	0.227	<0.034	0.311	<0.003	0.523	<0.0001	0.363	<0.001	0.422	<0.0001	0.127	<0.237
WHtR *z*-score	0.259	<0.015	0.327	<0.002	0.470	<0.0001	0.348	<0.001	0.421	<0.0001	0.141	<0.190
ET-1	1.000	—	0.944	<0.0001	0.806	<0.0001	0.897	<0.0001	0.845	<0.0001	0.922	<0.0001
IL-6	0.944	<0.0001	1.000	—	0.813	<0.0001	0.835	<0.0001	0.757	<0.0001	0.945	<0.0001
CRP	0.806	<0.0001	0.813	<0.0001	1.000	—	0.898	<0.0001	0.849	<0.0001	0.703	<0.0001
AGP	0.897	<0.0001	0.835	<0.0001	0.898	<0.0001	1.000	—	0.882	<0.0001	0.746	<0.0001
8-Isoprostane	0.845	<0.0001	0.757	<0.0001	0.849	<0.0001	0.882	<0.0001	1.000	—	0.662	<0.0001
8-OHdG	0.922	<0.0001	0.945	<0.0001	0.703	<0.0001	0.746	<0.0001	0.662	<0.0001	1.000	—

## Data Availability

The data used to support the findings of this study are available upon request.

## Supplementary Materials

Supplementary Materials A detailed methodology for ELISA has been described in supplementary materials.
