# Supplementary material for: Urinary Biomarkers of Inflammation and Oxidative Stress Are Elevated in Obese Children and Correlate with a Marker of Endothelial Dysfunction
Source: Oxid Med Cell Longev. 2019 Oct 16;2019:9604740. doi: 10.1155/2019/9604740 (PMC6817929; doi:10.1155/2019/9604740)
Supplement: Supplementary Materials — A detailed methodology for ELISA has been described in supplementary materials. [file 9604740.f1.pdf]

Supplemental materials

Measurement of low-grade inflammatory markers

Urinary inflammatory biomarkers such as interleukin-6 (IL-6; Cat # DY206), C-reactive protein (CRP; Cat # DY1707) and α-1-acid glycoprotein (AGP; Cat # DY3694) were measured by enzyme-linked sorbent assay (ELISA) (R&D systems, MN, USA) according to manufacturer instructions. Briefly, ELISA plates were prepared for IL-6, CRP, and AGP by adding 100 µl of capture antibody at the recommended working concentration and incubated overnight at room temperature. After incubation, plates were washed four times with 1x wash buffer and blotted in paper towels to remove any remaining wash buffer. Blocking was performed with 300 µl of reagent diluent and incubated 1 h at room temperature. Plates were washed with reagent diluent four times and 100 µl of sample or standards in duplicates, covered with adhesive films and incubated for 2 h at room temperature. After incubation plates were washed and incubated with 100 µl of the detection antibody (prepared in reagent diluent) for 2 h at room temperature. The detection antibody was aspirated and washed repeatedly four times with wash buffer. Plates were incubated in the dark avoiding the direct light with 100 µl of working dilution of freshly prepared streptavidin HRP for 20 min at room temperature. The same amount of substrate solution was added to each well and incubated for 20 min at room temperature after four washes. The reaction was stopped by adding 50 µl of stop solution, and plates were gently tapped to mix thoroughly. Plates were read at 450 nm with a wavelength correction. Standards of respective markers were used to calculate the levels of inflammatory markers. Since the urinary solutes concentration may differ by water reabsorption levels of the kidney, concentration of the inflammatory markers was normalized with corresponding urine creatinine levels (Cat # KGE005, R&D systems, MN, USA).

Endothelin-1 (ET-1; Cat # DET100, R&D systems, MN, USA) was measured as per the manufacturer's protocol. Briefly, 150 µl of assay diluent RD1-105 and 75 µl of standards or samples prepared in calibrator diluent RD5-48 added in each well and incubated for 1h at room temperature with shaking. Solutions were aspirated after incubation and washed four times with wash buffer. Endothelin-1 conjugate (200 µl) was added to each well and incubated in a shaker for 3h at room temperature. 200 µl of substrate solution was added to each well and incubated for 30min at room temperature after four washes. Place the plates on the benchtop and protect from direct light. Stop the reaction by adding 50 µl of stop solution to each well and read at 450 nm with a wavelength correction. The concentration of urine samples was calculated based on the computer generated four parameter logistic (4PL) curve fit. Results were normalized with urinary creatinine.

Assessment of Urinary oxidative stress markers

Two of the urinary oxidative stress markers namely, 8-isoprostane (Cat # ab175819, abcam, MA, USA) and 8-OHdG (Cat # 4380-192-K, R&D systems, MN, USA) were analyzed by ELISA. Briefly, analysis of 8-isoprostane was performed by competitive ELISA method. Each well 200 µl of all standards or samples were added (prepared in 1× sample dilution buffer) along with 100 µl of 1×-HRP conjugate except blank control wells. Equal volume to HRP conjugate, sample dilution buffer was used as maximum binding control. Standards and samples were incubated at room temperature for 2 h. After three washes with wash buffer plates were incubated with 200 µl of the TMB substrate to all wells. Plates were incubated at room temperature for 30 min, followed by 50 µl of stop solution added and read at 450 nm. The concentration of 8-isoprostane was normalized with urine creatinine values.

Urinary samples were processed for 8-hydroxy-2'-deoxyguanosine (8-OHdG) by ELISA analysis. In the pre-coated wells 25 µl of standards or samples along with the equivalent amount of anti-8 OHdG monoclonal antibody and incubated at room temperature for 1 h. An equal volume of assay diluent RD1-128 used as blank. Wash the plates with phosphate buffered saline Tween-20 four times, and 50 µl of HRP conjugated secondary antibody was added to each well except control well. Incubate the assay plate at room temperature for 1 h. After four repeated washes, 50 µl of pre-warmed TACS-sapphire solution added and incubated in the dark for 15 min at room temperature. The reaction was stopped by adding 50 µl of stop solution (0.2M HCl), mix well and read at 450 nm in BioTek ELISA plate reader. 8-OHdG was normalized with corresponding creatinine levels.
